# Dr. Ellis Vanderslice Ivey

**Published:** 1912-08

**Authors:** 


Dr. Ellis Vanderslice Ivey, a Bellevue interne, was killed in the D. L. & W. wreck at Corning, July 5.

